# Longitudinal trends and determinants of stunting among children aged 1–15 years

**DOI:** 10.1186/s13690-023-01090-7

**Published:** 2023-04-20

**Authors:** Senahara Korsa Wake, Temesgen Zewotir, Kebede Lulu, Yemane Hailu Fissuh

**Affiliations:** 1 Department of Statistics, College of Natural and Computational Sciences, Ambo University, Ambo, Ethiopia; 2 School of Mathematics, Statistics and Computer Science, College of Agriculture Engineering and Science, University of KwaZulu-Natal, Durban, South Africa; 3 Department of Statistics, College of Natural and Computational Sciences, Aksum University, Axum, Ethiopia

**Keywords:** Longitudinal data, Prevalence, Random effect, Stunting

## Abstract

**Background:**

Stunting increases morbidity and mortality, hindering mental development and influencing cognitive capacity of children. This study aimed to examine the trends and determinants of stunting from infancy to middle adolescence in four countries: Ethiopia, India, Peru, and Vietnam.

**Methods:**

A 15-year longitudinal data on the trends of stunting were obtained from the Young Lives cohort study. The study includes 38,361 observations from 4 countries. A generalized mixed-effects model was adopted to estimate the determinant of stunting.

**Results:**

The patterns of stunting in children from aged 1 to 15 years have declined from an estimated 30% in 2002 to 20% in 2016. Stunting prevalence varied among four low- and middle-income countries with children in Ethiopia, India, and Peru being more stunted compared to children in Vietnam. The highest stunted was recorded in India and the lowest was recorded in Vietnam. In all four countries, the highest prevalence of severe stunting was observed in 2002 and moderate stunting was observed in 2006. Parents’ education level played a significance role in determining a child stunting. Children of uneducated parents were shown to be at a higher risk of stunting.

**Conclusion:**

Disparities of stunting were observed between- and within-country of four low- and middle-income with the highest prevalence recorded in low-income country. Child stunting is caused by factors related to child’s age, household wealth, household size, the mother’s and father’s education level, residence area and access to save drinking water.

**Supplementary Information:**

The online version contains supplementary material available at 10.1186/s13690-023-01090-7.

## Background

The Sustainable Development Goals and the 2030 Agenda for Sustainable Development both call for reducing disparities in health and leaving no one behind [1, 2]. Health disparities are linked to social disparities in nutrition via complex pathways that include policies addressing health and nutrition; the quality and quantity of food available and consumed; access to and affordability of nutritious foods of high quality; and individuals’ and populations’ living conditions and circumstances. To effectively plan, create, and implement public health nutrition policies, strategies, and programs, the evidence base on health disparities in nutrition must be expanded [3].

Stunting, wasting, and underweight have often been used to measure the prevalence of under-nutrition in children. Stunting is the delayed growth and development that children endure as a result of poor nutrition, frequent infection, and insufficient psychosocial stimulation [4]. Children are considered stunted if their height-for-age is more than two standard deviations below the WHO Child Growth Standards median [5, 6]. Stunting raises morbidity and mortality risks in children, and also hinders mental development, and influence the cognitive ability of children [5, 7–9]. It is a physical sign of persistent childhood malnutrition that is clearly visible and quantified [6].

In recent decades, there has been significant success in lowering child stunting prevalence [3, 6, 9, 10]. However, between-country disparities in the prevalence of stunting reduction are widely observed. Africa and Asia suffer the greatest burden of a child stunting [11]. Stunting prevalence, for example, has decreased by two-thirds in upper-middle-income nations since 2000, whereas it remains high in low and lower-middle-income nations [10, 12, 13].

The Sustainable Development Goals of the United Nations have identified stunting, along with other nutrition indicators, as the primary focal areas for eradicating global malnutrition [14]. The disparity in worldwide progress in child stunting provides an appropriate scenario for testing the hypothesis in child stunting trends. In this regard, it is critical to investigate the major causes and trends of stunting so that individual nations may learn what works and develop customized policies and initiatives.

The objective of this study was, therefore, to investigate the patterns and determinants of stunting from infancy to middle adolescence in four countries: Ethiopia, India, Peru, and Vietnam.

## Materials and methods

### Data sources and settings

Longitudinal data on the prevalence of stunting were obtained from the Young Lives cohort study. The Young Lives study is a 15-year longitudinal study that looks at the changing nature of childhood poverty in Ethiopia, India, Peru, and Vietnam. The Young Lives cohort study thus collects data from these countries at both child-level and household-level to understand the causal effect of childhood poverty. Children from poor household were purposively over-sampled [15].

For sample selection, a multistage sampling approach was adopted, with the first stage including the selection of 20 sentinel stations from each nation. Following the selection of 20 sentinel sites, households with children, on average, one-year age groups were chosen at random. Finally, 100 households with, on average, a one-year-old child were selected randomly from each sentinel site [16]. A longitudinal anthropometric measurements of children were collected at the years 2002, 2006, 2009, 2013, and 2016 [17]. The sample and sampling procedures adopted in the Young Lives cohort study were addressed in detail elsewhere [18–23].

### The study variables

The stunting status of children was a categorical outcome variable. It was measured longitudinally on five measurement occasions from 2002 to 2016. Children with height-for-age z-scores (HAZ) greater than or equal to -2 were categorized as not stunted, moderately stunted if their HAZ were between − 3 and − 2, and severely stunted if their HAZ were less than − 3 [3, 15, 24, 25]. The Independent variables considered as determinants of stunting were gender, age, residence area (rural and urban), father’s level of education, father’s age, mother’s level of education, mother’s age, household size, wealth index, and access to safe drinking water. In the Young Lives study, the wealth index is the major measure of households’ socioeconomic status [19]. It is the average of housing quality, access to services, and consumer durable ownership. This average yields a number between 0 and 1, with a higher wealth index reflecting a better socioeconomic status [15].

### Ethical approval

In this study, the secondary data from the UK archival sources were used. The UK data are publicly available and can be accessed from this link http://www.younglives.org.uk/ by contacting the UK program team using personal accounts and providing the reason for the request. After reviewing the abstract of our study, we were given permission to use the data.

### Data analysis methods

#### Generalized linear mixed-effect model (GLMM)

In a current longitudinal data, individuals in the study are followed over a period of time and, for each individual, data were collected at five time points from aged 1 to 15 years. One of the mixed-effects classes commonly used for analyzing longitudinal binary data is the generalized linear mixed model. Consider a binary longitudinal outcome of stunting ($$ {y}_{ij}$$, $$ i,\dots ,n;j=1,\dots ,{n}_{i}$$) taking only two possible values (not stunted coded as 0 and stunted coded as 1). In this regard, a general GLMM for binary longitudinal outcome can be written as [26]

$$ \text{log}\left(\frac{{\mu }_{ij}}{1-{\mu }_{ij}}\right)={\varvec{x}}_{ij}^{T}\beta +{\varvec{z}}_{ij}^{T}{\varvec{b}}_{i}$$

where ${\mu }_{ij}=E\left({y}_{ij}|\beta ,{b}_{i}\right)$, ${b_i} \sim N(0,D)$, ${\varvec{x}}_{i}$ and ${\varvec{z}}_{i}$ are vectors containing fixed and random covariates, respectively, $\beta$ is a vector of fixed effect and D is a covariance matrix. A GLMM is typically based on the likelihood method of statistical inference [26, 27].

The fixed variables (${\varvec{x}}_{\varvec{i}}$) included in a GLMM are: gender, age, residence area, father’s level of education, father’s age, mother’s level of education, mother’s age, household size, wealth index, and access to safe drinking water. Children (Children Id) were considered as random variable (${\varvec{z}}_{\varvec{j}}$) in this study. The outcome variable (stunting) was reclassified into two categories: stunted and not stunted, with moderately stunted and severely stunted being merged into the term stunted. Hence, the data were analyzed by using a binary longitudinal outcome in GLMM.

The data were analyzed using Statistical Analysis Systems SAS 9.4 version. Modeling categorical response variables with random effects is a major use of the PROC GLIMMIX in SAS statistical procedure. The PROC GLIMMIX provides the capability to model binary (not-stunted/stunted, 0/1) outcome, including random effects and correlated errors.

## Results

### Characteristics of the study participants

**Fig. 1:**
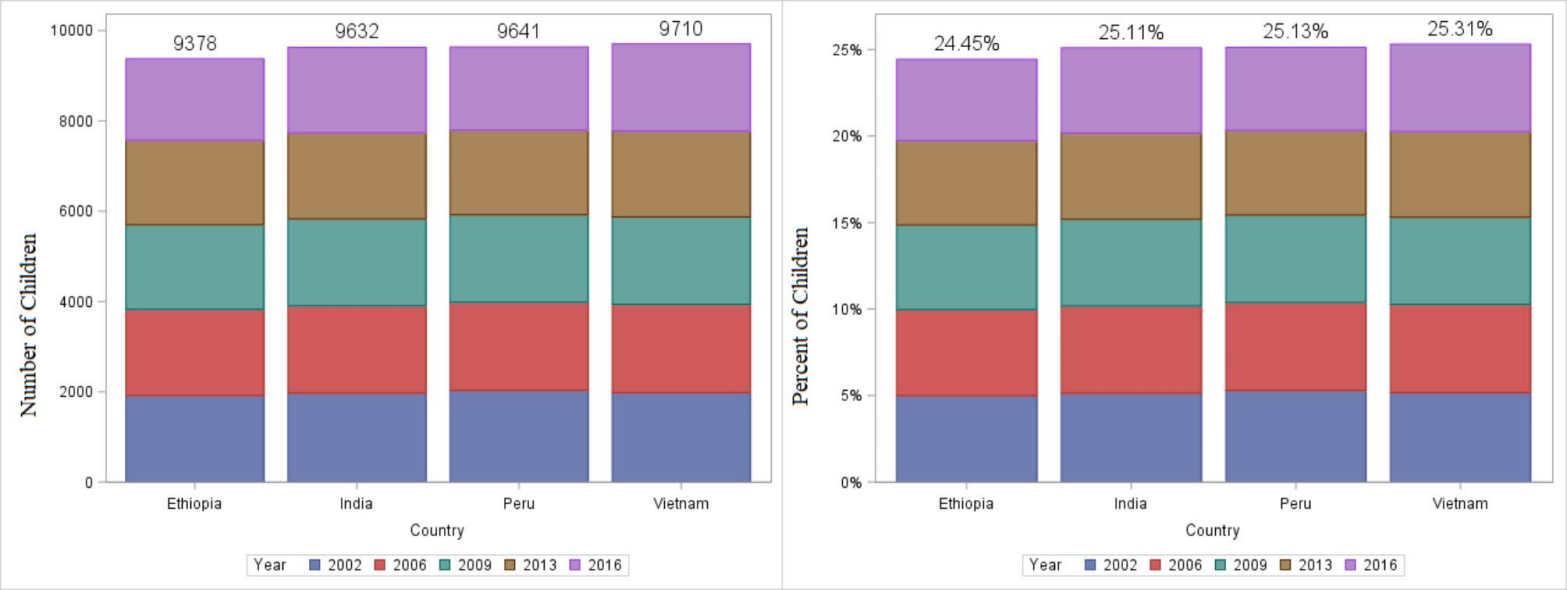
The number and percent of children included in the study from Ethiopia, India, Peru, and Vietnam

Table 1 shows the background characteristics of the children, their parents, and the countries from which the children were chosen. In all, 38,361 observations were available from 4 countries. Of these observations, 9378, 9632, 9641, and 9710 were conducted in Ethiopia, India, Peru, and Vietnam, respectively. The percentage of observations (children chosen) from each country was nearly identical, the minimum was 9378(24.45%) from Ethiopia and the maximum was 9710(25.31%) from Vietnam (Fig. 1). The majority of the children in the sample were from rural areas, and more than half of them were males. In 2002, the majority of children’s mothers (40.3%) were uneducated. However, the percentage of uneducated mothers has decreased over time from 40.3% to 2002 to 24.6% in 2016. Similarly, the percentage of uneducated fathers fell from 32.6% to 2002 to 12.6% in 2016 (Table 1).

**Table 1:** Background Characteristics of children in Ethiopia, India, Peru, and Vietnam

Characteristics			Year of survey				
			2002	2006	2009	2013	2016
Child’s sex	Male	n	4091	4040	3990	3935	3900
		%	51.8	52.1	52.0	52.1	52.1
	Female	n	3814	3711	3682	3619	3579
		%	48.2	47.9	48.0	47.9	47.9
Area of residence	Urban	n	2976	2925	2940	3015	3044
		%	37.6	37.7	38.3	39.9	40.7
	Rural	n	4929	4826	4715	4539	4434
		%	62.4	62.3	61.5	60.1	59.3
Access to safe drinking water	No	n	3999	3340	2771	2569	2198
		%	50.6	43.1	36.2	34.0	29.5
	Yes	n	3901	4411	4892	4981	5257
		%	49.4	56.9	63.8	66.0	70.5
Father’s education level	Uneducated	n	2295	1464	1232	870	809
		%	32.6	21.0	18.4	13.2	12.6
	Primary school	n	2462	3013	2998	3010	2713
		%	34.9	43.2	44.7	45.8	42.3
	Secondary school	n	1749	1765	1734	1750	1849
		%	24.8	25.3	25.9	26.6	28.8
	Diploma and above	n	521	553	547	617	676
		%	7.4	7.9	8.2	9.4	10.5
	Adult and Religious education	n	21	176	189	324	364
		%	0.3	2.5	2.8	4.9	5.7
Mother’s education level	Uneducated	n	3115	2357	2004	1844	1757
		%	40.3	31.1	27.2	25.4	24.6
	Primary school	n	2660	3113	3212	3133	2924
		%	34.4	41.1	43.6	43.1	41.0
	Secondary school	n	1522	1524	1510	1538	1628
		%	19.7	20.1	20.5	21.2	22.8
	Diploma and above	n	414	412	432	462	525
		%	5.4	5.4	5.9	6.4	7.4
	Adult and Religious education	n	19	163	215	287	300
		%	0.2	2.2	2.9	4.0	4.2
The four Low- and middle-income countries	Ethiopia	n	1917	1908	1877	1871	1805
		%	24.3	24.6	24.5	24.8	24.1
	India	n	1970	1937	1924	1907	1894
		%	24.9	25.0	25.1	25.2	25.3
	Peru	n	2035	1950	1937	1876	1843
		%	25.7	25.2	25.2	24.8	24.6
	Vietnam	n	1983	1956	1934	1900	1937
		%	25.1	25.2	25.2	25.2	25.9

**Fig. 2:**
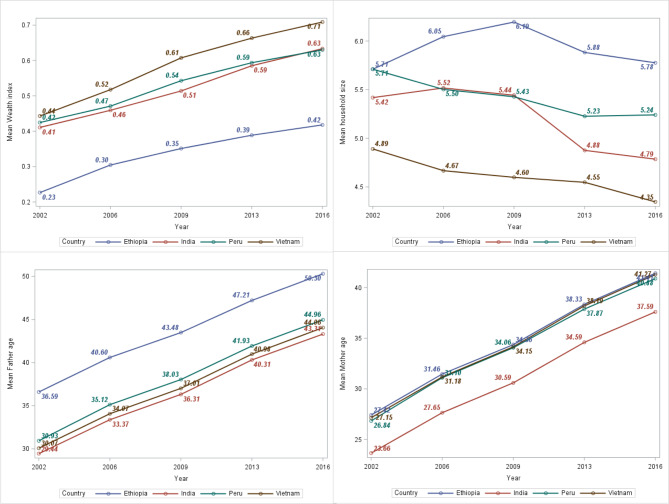
The mean trends of father’s age, mother’s age, wealth index, and household size in Ethiopia, India, Peru, and Vietnam

Figure 2 depicts the graphical exploration of the characteristics of numeric variables such as father’s age, mother’s age, wealth index, and household size. From Fig. 2, it can be seen that Vietnam had the highest wealth index and the lowest household size throughout all the study periods. In contrast, Ethiopia had the lowest wealth index and the largest household size from 2002 to 2016. In all study periods from 2002 to 2016, the parents (father and mother) of Indian children had the lowest average ages, while the parents of Ethiopian children had the highest average ages.

### Height-for-age

**Fig. 3:**
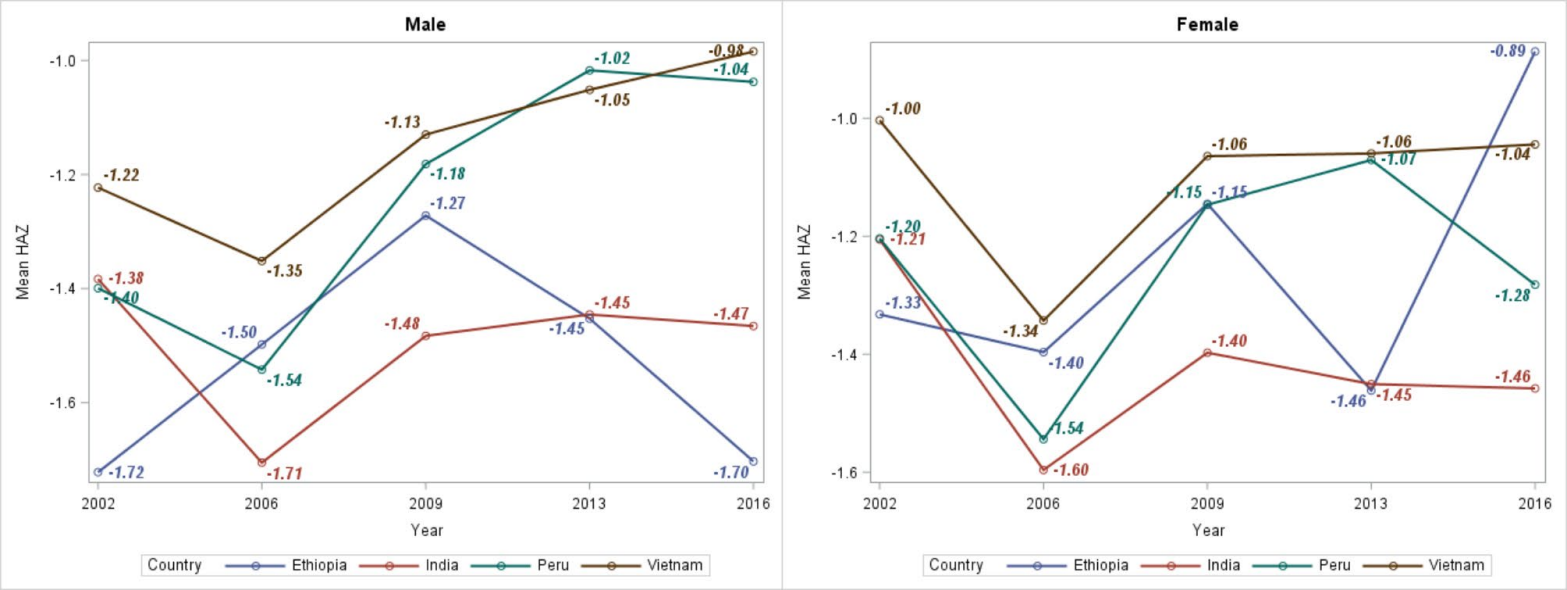
Mean height-for-age z-score of children in Ethiopia, India, Peru, and Vietnam

From 2002 to 2016, height-for-age z-score (HAZ) values were computed for each child in four study countries to assess the trends and prevalence of stunting. Figure 3 shows trends in the mean HAZ of children in the four study countries. The minimum (-1.72) and maximum (-0.98) estimated mean HAZ were recorded in Ethiopian males and females, respectively. Children in Vietnam had approximately the highest mean HAZ in both genders, except in 2016, where Ethiopian females had the highest estimated mean HAZ (-0.89).

### Between-country patterns of stunting prevalence from 2002 to 2016

**Fig. 4:**
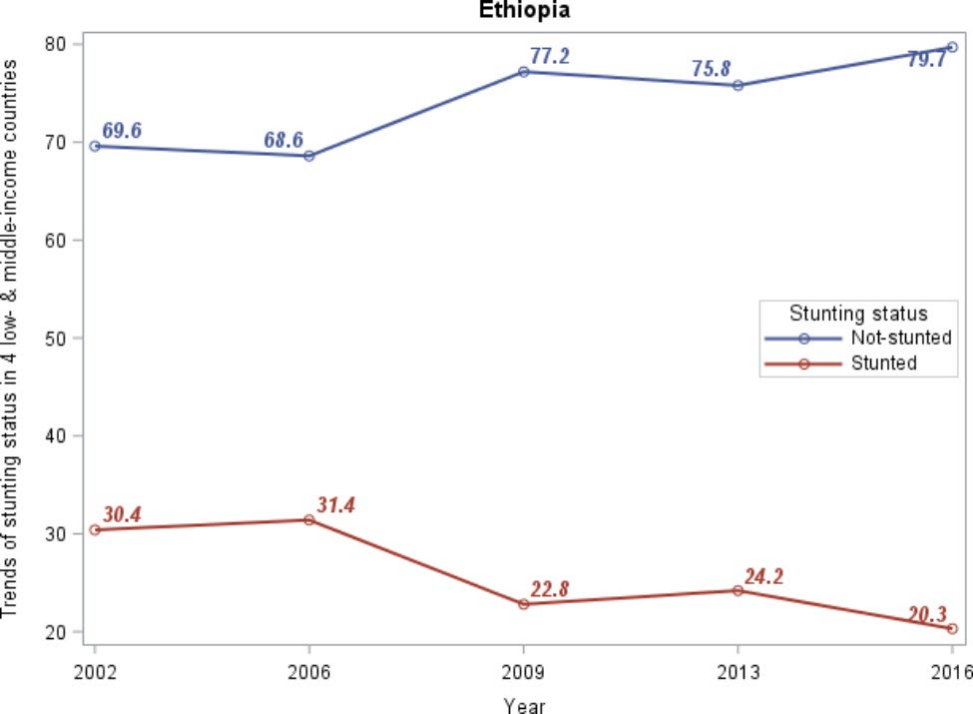
Trends of stunting in four low- and middle-income countries

**Fig. 5:**
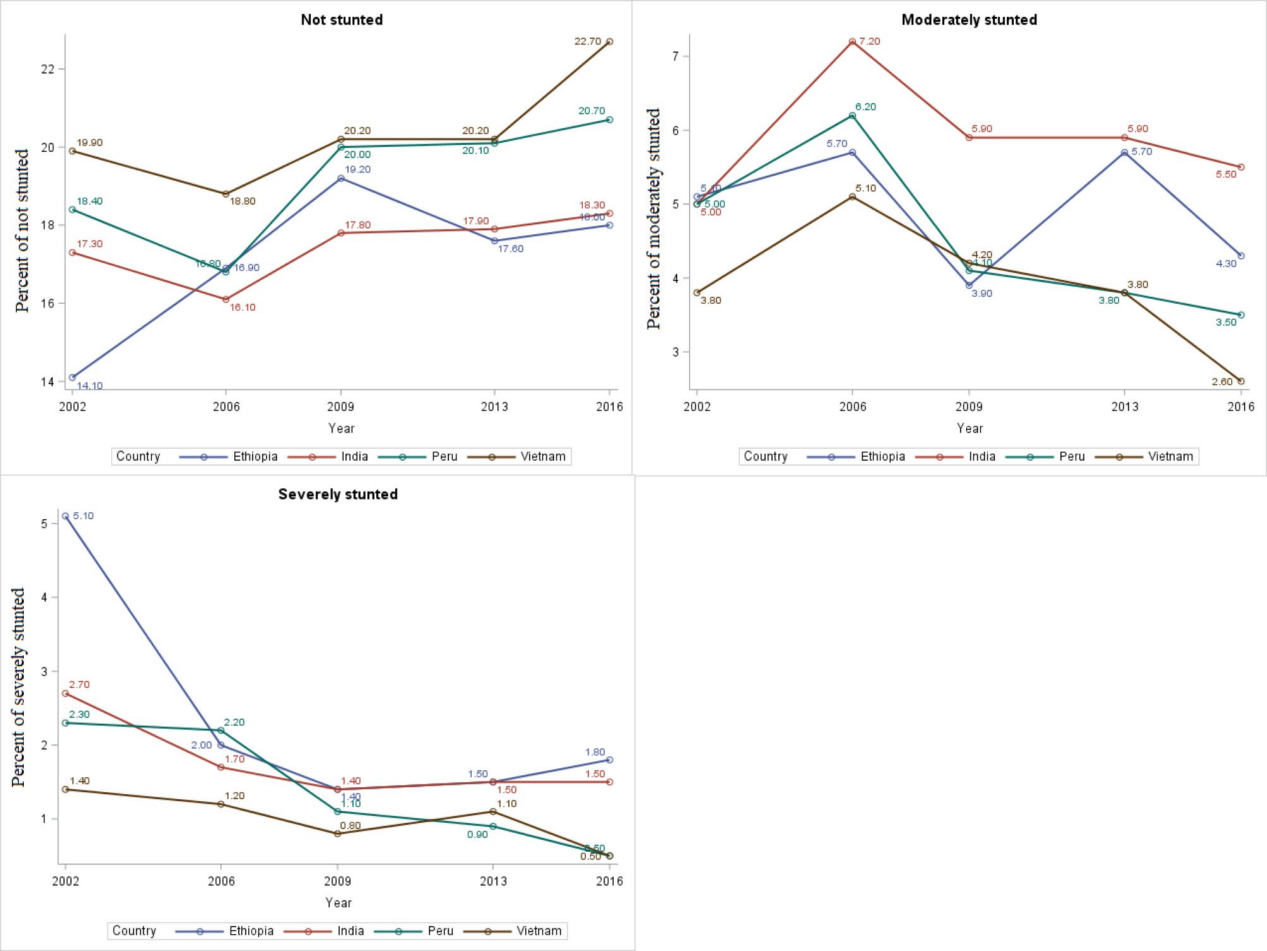
Between-country trends of stunting status among four low- and middle-income countries from 2002 to 2016

The within- and between-country status of stunting prevalence was presented in Table 2; Fig. 4, respectively. From the visual inspection of the between-country trends of stunting prevalence displayed in Fig. 4, the trends of stunting in children from 2002 to 2016 have declined from an estimated 30% in 2002 to 20% in 2016. In fact, the four countries have observed reductions in stunting prevalence. However, the greatest decline occurred in Vietnam to 2.6% in moderately stunted and to 0.5% in severely stunted (Fig. 5).

From the results of within-county prevalence of stunting status presented in Table 2, for all four countries, the highest prevalence of severe stunting was observed in 2002 and moderate stunting was observed in 2006. For instance, the prevalence of severe stunting in 2002 was 20.9%, 10.8%, 9.1%, and 5.7% in Ethiopia [23], India, Peru, and Vietnam, respectively. On the other hand, the highest prevalence of moderate stunting observed in 2006 was 23.2%, 28.7%, 24.5%, and 20.4% in Ethiopia, India, Peru, and Vietnam, respectively (Table 2). Accordingly, the lowest prevalence of severe stunting was observed in 2009 for both Ethiopia and India (5.7% and 5.5%, respectively) and in 2016 for Peru and Vietnam (both 2%).

**Table 2:** Within-country (Ethiopia, India, Peru, and Vietnam) prevalence of stunting status

Country	Stunting		Prevalence, n(%)				
			2002	2006	2009	2013	2016
Ethiopia	Not stunted	n	1112	1310	1474	1329	1345
		%	58	68.7	78.5	71	74.5
	Moderately stunted	n	405	442	296	431	324
		%	21.1	23.2	15.8	23	18
	Severely stunted	n	400	156	107	111	136
		%	20.9	8.2	5.7	5.9	7.5
India	Not stunted	n	1365	1246	1368	1350	1368
		%	69.3	64.3	71.1	70.8	72.2
	Moderately stunted	n	392	556	450	444	415
		%	19.9	28.7	23.4	23.3	21.9
	Severely stunted	n	213	135	106	113	111
		%	10.8	7	5.5	5.9	5.9
Peru	Not stunted	n	1458	1303	1537	1520	1547
		%	71.6	66.8	79.3	81	83.9
	Moderately stunted	n	392	478	317	289	259
		%	19.3	24.5	16.4	15.4	14.1
	Severely stunted	n	185	169	83	67	37
		%	9.1	8.7	4.3	3.6	2
Vietnam	Not stunted	n	1570	1461	1547	1528	1700
		%	79.2%	74.7	80	80.4	87.8
	Moderately stunted	n	300	399	326	288	198
		%	15.1	20.4	16.9	15.2	10.2
	Severely stunted	n	113	96	61	84	39
		%	5.7	4.9	3.2	4.4	2

**Fig. 6:**
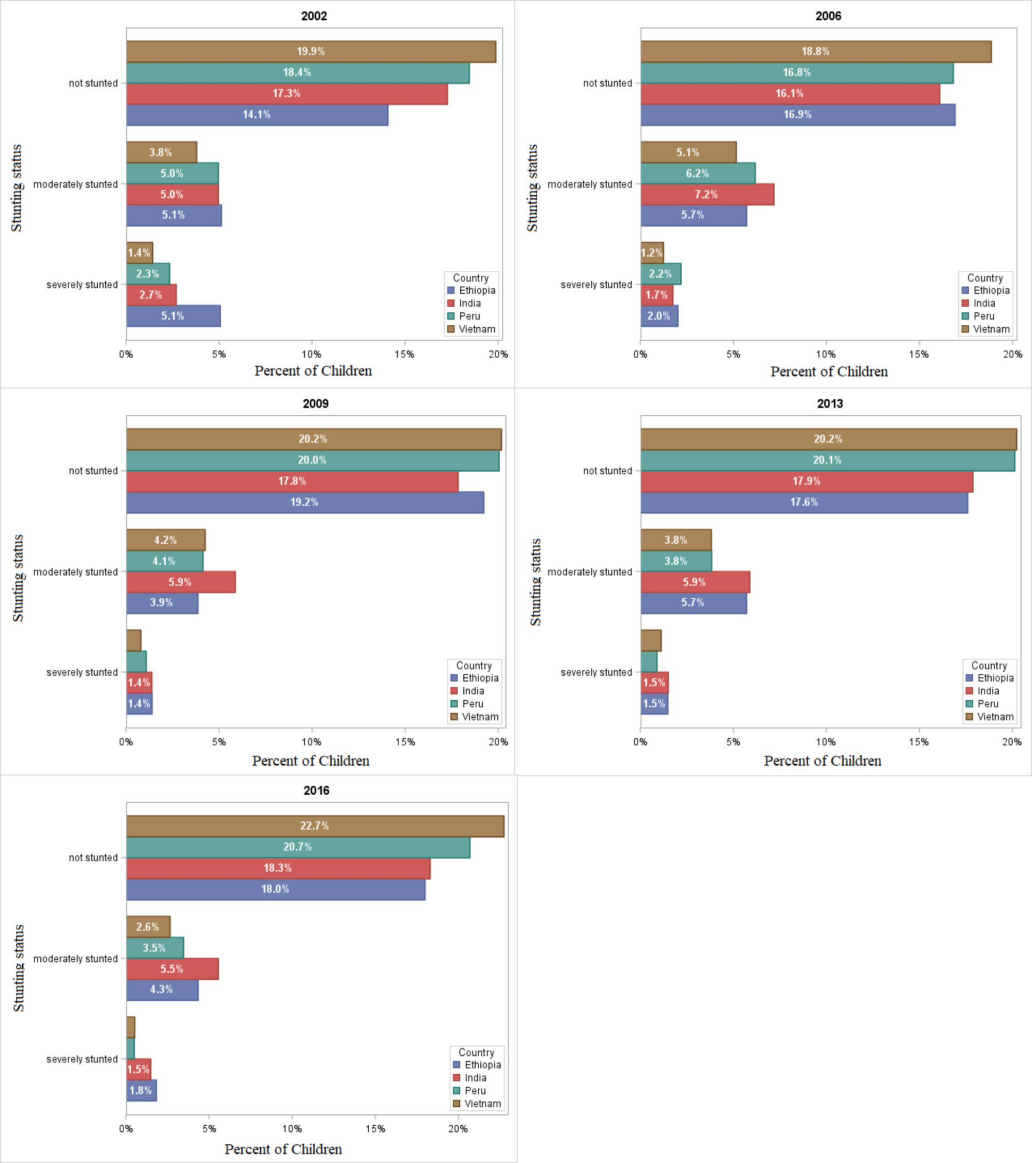
Between-country prevalence of stunting among four low- and middle-income countries from 2002 to 2016

Figures 6 and 5 depict the prevalence and patterns of stunting status from 2002 to 2016. The percentages of between-country prevalence were derived using the sum of children numbers affected in each country divided by the total children in four countries. The percentages of within-country prevalence, on the other hand, were calculated by dividing the total number of children exposed in each country by the total number of children in the corresponding country.

### Results of generalized linear mixed-effects model

Stunting determinants were chosen by first including all considered factors into the model and then evaluating their significance. Subsequently, those factors that were statistically significant in determining a child stunting were considered and interpreted. In this regard, factors such as a child’s country, a child’s age, household size, wealth index, child’s sex, residence area, father’s education level, and mother’s education level were statistically significant determinants of stunting. However, father’s age and mother’s age were not statistically significant determinants of stunting. The significance tests of variables are presented in Table 3.

**Table 3:** The fixed effects significance tests in determinants of stunting

Effect	Num DF	Den DF	F Value	Pr > F
Country	3	32,616	30.64	< 0.0001
Child’s age	4	32,616	46.21	< 0.0001
Mother’s age	2	32,616	0.72	0.4859
Father’s age	2	32,616	0.07	0.9349
Household size	1	32,616	8.59	0.0034
Wealth index	2	32,616	52.11	< 0.0001
Child’s sex	1	32,616	36.00	< 0.0001
Residence area	1	32,616	50.83	< 0.0001
Father’s education level	4	32,616	11.59	< 0.0001
Mother’s education level	4	32,616	25.16	< 0.0001

Table 4 depicts the estimated odds ratios of factors related to parents’ and children’s characteristics in determining a child’s stunting. The findings of the study revealed that there were disparities in stunting prevalence in four low- and middle-income countries; Ethiopia, India, Peru, and Vietnam. Compared to children in Vietnam, children in Ethiopia, India, and Peru were more stunted (Ethiopia: OR = 1.53, 95% CI: 1.21–1.93, p = 0.0003, India: OR = 2.33, 95% CI: 1.89–2.88, p < 0.0001, Peru: OR = 2.57, 95% CI: 2.04–3.24, p < 0.0001). In terms of ages, children were more stunted at five years old (OR = 1.27, 95% CI: 1.14–1.42, p < 0.0001) and less stunted at eight (OR = 0.61, 95% CI: 0.53–0.69, p < 0.0001), twelve (OR = 0.76, 95% CI: 0.65–0.89, p = 0.0006), and fifteen (OR = 0.55, 95% CI: 0.46–0.66, p < 0.0001) years old.

Girls and boys are not equally likely to be stunted in these four low- and middle-income countries, but in these countries, stunting afflicts fewer girls (OR = 0.65, 95% CI: 0.56–0.75, p < 0.0001) than boys. This suggests that girls had a 35% lesser odds of stunting compared to boys. Children in rural households were more than 1.82 times (OR = 1.82, 95% CI: 1.54–2.15, p < 0.0001) as likely to be stunted as children in urban households.

Regarding parents-related factors, children from educated parents (mother and father), children from high wealth index households, and children from low household sizes were associated with lower odds of stunting. For instance, children born to mothers with a primary school, secondary school, and diploma and above education level had 0.72 (95% CI: 0.62–0.84, p < 0.0001), 0.35 (95% CI: 0.28–0.4, p < 0.0001), and 0.17 (95% CI: 0.11–0.27, p < 0.0001) times less likely to be stunted than children from uneducated mothers, respectively. Similarly, children born to fathers with a secondary school, and diploma and above education level had 0.53 (95% CI: 0.43–0.66, p < 0.0001), 0.499 (95% CI: 0.357–0.699, p < 0.0001), and 0.17 (95% CI: 0.11–0.27) times less likely to be stunted than children from uneducated mothers, respectively. Children from the medium and high wealth index households were less than 0.68 (95% CI: 0.61–0.76, p < 0.0001) and 0.43 (95% CI: 0.36–0.50, p < 0.0001) times as likely to be stunted as children from the low wealth index households, respectively.

**Table 4:** The fixed effects factors related to parents’ and children’s characteristics

Effect	Estimate	SE	OR	95% CI	P-value
**Country (ref. = Vietnam)**					
Ethiopia	0.4261	0.1183	1.531	1.214–1.931	0.0003
India	0.8468	0.1069	2.332	1.891–2.876	< 0.0001
Peru	0.9445	0.1181	2.572	2.04–3.241	< 0.0001
**Child’s age in year (ref. = age one)**					
Age five	0.2384	0.0554	1.269	1.139–1.415	< 0.0001
Age eight	-0.503	0.0661	0.605	0.531–0.688	< 0.0001
Age twelve	-0.276	0.0799	0.759	0.649–0.887	0.0006
Age fifteen	-0.591	0.0915	0.554	0.463–0.663	< 0.0001
**Mother’s age in year (ref. =≤29 age)**					
30≤ age ≤36	0.07984	0.0665	1.083	0.951–1.234	0.2301
≥37 age	0.09663	0.1016	1.101	0.903–1.344	0.3413
**Father’s age in year (ref. = ≤34 age)**					
35≤ age ≤41	0.00028	0.0654	1	0.88–1.137	0.9966
> 41 age	0.02459	0.0969	1.025	0.848–1.239	0.7996
**Household size in number (ref.=≤5 household size)**					
>5 household size	0.1478	0.0504	1.159	1.05–1.28	0.0034
**Wealth index (ref. = low)**					
Medium	-0.3833	0.0575	0.682	0.609–0.763	< 0.0001
High	-0.8559	0.0839	0.425	0.36–0.501	< 0.0001
**Child’s sex (ref. = Boy)**					
Girl	-0.4372	0.0729	0.646	0.56–0.745	< 0.0001
**Residence area (ref. = Urban)**					
Rural	0.5994	0.0841	1.821	1.544–2.147	< 0.0001
**Father education level (ref. = uneducated)**					
Adult and religious education	0.1172	0.1465	1.124	0.844–1.498	0.4235
Primary school	-0.0896	0.0781	0.914	0.785–1.065	0.2512
Secondary school	-0.6365	0.1092	0.529	0.427–0.655	< 0.0001
Diploma and above	-0.6942	0.1718	0.499	0.357–0.699	< 0.0001
**Mother education level (ref. = uneducated)**					
Adult and religious education	-0.0921	0.1661	0.912	0.659–1.263	0.5793
Primary school	-0.3241	0.0784	0.723	0.62–0.843	< 0.0001
Secondary school	-1.0428	0.1206	0.352	0.278-0.4	< 0.0001
Diploma and above	-1.75	0.2174	0.174	0.113–0.266	< 0.0001

## Discussion

This study covered four low- and middle-income countries of the Young Lives study area; namely Ethiopia, India, Peru, and Vietnam. The Young Lives data are well-suited for studying the longitudinal dynamics of child-related variables as well as the influence of children’s early-life situations on their later outcomes. As a result, the data derived from the Young Lives study and used in this study contained both country-specific and statistically sound information.

The current study covered only four low- and middle-income countries and reported on the patterns, prevalence, and determinants of stunting from 2002 to 2016. Countries are expected to identify their contributions and set their own objectives in order for the global stunting target to be met. The ability to translate the global aim into individual national targets is reliant on nutrition profiles, risk factor trends, demographic changes and executing nutrition policies, and the level of health system development [11]. Therefore, the findings of this study insight into the stunting prevalence in these countries that are used to show the countries’ contribution to the global stunting target.

In this study, the within- and between-country status of stunting prevalence was examined. The trends of stunting prevalence among children aged 1 to 15 years in four low- and middle-income countries were declined from 30 to 20% (Fig. 4). According to Onis et al. [28] study on the prevalence and trends of stunting among preschool children in developing countries, stunting prevalence declined from 47% to 1980 to 33% in 2000 (by 40 million). Despite the overall decrease in stunting, progress was heterogeneous across countries. The highest moderate stunted was recorded in India during all the study periods, whereas Vietnam had the lowest prevalence except in 2009. Ethiopia recorded the highest percentage of severe stunting (5.1%) in 2002 and the lowest percentage of moderate stunting (3.9%) in 2009 compared with that of India, Peru, and Vietnam. This result is similar to the findings of Astatkie [23], who utilized the same dataset to investigate dynamics of stunting in Ethiopia using both younger and older cohort data. He noted that the highest cross-sectional prevalence of severe stunting in younger children was observed in Round 1 (2002).

A generalized linear mixed-effects model was adopted to estimate the determinant of stunting from 2002 to 2016. The basic model contained the factors country, child’s age, mother’s age, father’s age, household size, wealth index, child’s sex, residence area, father’s education level, and mother’s education level as fixed effects and children as a random effect.

Stunting prevalence varied among four low- and middle-income countries with children in Ethiopia, India, and Peru being more stunted compared to children in Vietnam. The study identified that a child’s age was a significant factor in determining a child stunting. At ages 8, 12, and 15 years, children were less likely to be stunted than at age 1 year. However, Children were more likely to be stunted at five years of age than at one year of age. This result is consistent with the previous studies [23, 29–31].

The study showed that girls were at a lesser risk for stunting than boys. This was confirmed in several previous studies [23, 29, 32–37]. In contrast, previous studies found no significant variation in stunting based on gender [38, 39]. Furthermore, the findings indicated that the mother’s education level and the father’s education level also played a role in determining child stunting. One likely reason is that more education implies literacy, which allows parents to obtain more health information. Better feeding patterns and nutrition status in Ugandan children have been linked to maternal literacy and education [40]. Children of uneducated parents were shown to be at a higher risk of stunting. This result is consistent with earlier findings [31, 33, 34, 41, 42]. Conversely, a study in Uganda noted that no significant effect of education level on stunting [38], while other studies found an inverse relationship between education level and stunting [43, 44]. Berhane et al. reported that more educated mothers provided a more diverse diet for their children. Furthermore, greater education opens the door to a higher income, enhancing household wealth [32].

This study noted that children of households with a medium and high wealth index had a much lower risk for stunting than low wealth index households. Several previous studies found that children of a rich household were related to a lower risk of stunting [33, 34, 42, 45]. A study of Ethiopian children demonstrated that low socioeconomic status may predispose children to stunting [46]. Residence area is another environmental factor that impacts food security and thus could changes child stunting. Children in rural households were also found to be at a higher risk of stunting as compared to urban households. Furthermore, the study showed that households of several sizes living together increased the risk of stunting in those children. It is evident that the food available must be distributed to all household members, which raises the risk of under-nutrition in less resourceful households. Living many household sizes together puts a greater strain on caregivers and household resources as compared to a singleton [37].

Several previous studies were used cross-sectional data to evaluate the nutrition status of children [47–50]. It is rational that cross-sectional studies are restricted in quantifying individual changes over time. One of the strength of this study is that it utilized longitudinal data to examine the prevalence of stunting over time from 2002 to 2016 in Ethiopia, India, Peru and Vietnam. Longitudinal studies are more successful and have higher statistical power than cross-sectional studies in evaluating changes over time [51, 52]. There are also some limitations to the present analyses. This study was limited to four low- and middle-income countries; hence, the results present in this study may not reflect all children in low- and middle-income countries. Another limitation of the study is that the Young Lives study employed a purposive sampling technique to select sentinel sites from which children under the study can be chosen. This may limit to generalize the study results. Despite these limitations, the current findings provide a good foundation for monitoring stunting levels and changes.

## Conclusions

In conclusion, the pattern of stunting among children aged 1 to 15 years has reduced in four low- and middle-income countries. Despite the overall decrease, progress of decrement was heterogeneous across countries. The highest stunted was recorded in India during all the study periods, and approximately the lower was recorded in Vietnam. Ethiopia recorded the highest percentage of severe stunting in 2002 and the lowest percentage of moderate stunting in 2009. Child stunting is caused by factors associated with child’s age, household wealth, household size, the mother’s and father’s education level, residence area and access to save drinking water. Policies and interventions should focus on parents’ education and access to save drinking water, as these are the most powerful factors.

## Electronic supplementary material

Below is the link to the electronic supplementary material.

Supplementary Material 1

## Data Availability

The Young Lives data are publicly available and can be accessed from http://www.younglives.org.uk/.
