# Supplementary material for: Longitudinal trends and determinants of stunting among children aged 1–15 years
Source: Arch Public Health. 2023 Apr 20;81:60. doi: 10.1186/s13690-023-01090-7 (PMC10116743; doi:10.1186/s13690-023-01090-7)
Supplement: Supplementary file 1 — Supplementary Material 1 [file 13690_2023_1090_MOESM1_ESM.docx]

**Aims and Scope statement**

**1. What is known?**

Stunting, wasting, and underweight have often been used to measure the prevalence of under-nutrition in children. Stunting is the delayed growth and development that children endure as a result of poor nutrition, frequent infection, and insufficient psychosocial stimulation. To address health inequality, a better knowledge of life-course changes is essential. The use of life-course perspectives on the formation of inequalities permits us to investigate the dynamic link between early-life circumstances and later repercussions, as well as to determine the best timing in terms of when circumstances and events have the most impact. Children's stunting is characteristics of physical, psychological, biological, and sociological natural events. When sufficient allowances are made for variations in genetic ability, average values of children's height accurately represent the condition of a nation's public health and the average nutritional status of its people, particularly in developing countries. For instance, children from low-income families have poorer health than children from higher-income families. Due to diverse ethnicity and cultural family backgrounds, heights of healthy children differ in different areas of the world. As a result, research into differences in stunting changes over time and across countries can aid in identifying differences in childhood standards of living. Several studies were conducted as part of the Young Lives study to develop a quantitative and qualitative child-focused development study that captures the development status of children through interactive processes influenced by biological, psychological and environmental interactions. To date, there is no evidence of longitudinal trends and variations in stunting among children in Ethiopia, India, Peru, and Vietnam from age 1 to 15 years and no such study has been carried out for a given site. This study is aimed to investigate variations and patterns in stunting among children in four low-and middle-income countries.

**2. What does the study add?**

The Young Lives cohort study is the first multi-country longitudinal cohort study of its type
carried out in four low- and middle-income countries to enhance knowledge of the causes and effects of childhood poverty, as well as to investigate how policies impact children's well-being, in order to better guide future policy formulation and to better focus child welfare initiatives. In this study we examined the longitudinal trends and determinants of stunting among children aged 1-15 years in four low- and middle-income countries. Stunting prevalence varied among four low- and middle-income countries with children in Ethiopia, India, and Peru being more stunted compared to children in Vietnam. Therefore, this study adds to the literature by estimating variations in stunting changes among children in low- and middle-income countries. A clear understanding of stunting variations is important for finding key possibilities to promote healthy growth in early life of children in low- and middle-income countries.

**3. What are implications for clinical practice, public health and / or research?**

In conclusion, the pattern of stunting among children aged 1 to 15 years has reduced in four low- and middle-income countries. Despite the overall decrease, progress of decrement was heterogeneous across countries. The highest stunted was recorded in India during all the study periods, and approximately the lower was recorded in Vietnam. Ethiopia recorded the highest percentage of severe stunting in 2002 and the lowest percentage of moderate stunting in 2009. Based on these findings, policies and interventions should focus on countries with the highest prevalence of stunting.
